# Serotonin Signaling and Macrophage Subsets in Goldfish Gills: Unraveling the Neuroimmune Network for Gill Homeostasis

**DOI:** 10.3390/life15050751

**Published:** 2025-05-07

**Authors:** Manal T. Hussein, Giacomo Zaccone, Marco Albano, Alessio Alesci, Sebastian Marino, Rasha Alonaizan, Doaa M. Mokhtar

**Affiliations:** 1 Department of Cell and Tissues, Faculty of Veterinary Medicine, Assiut University, Assiut 71526, Egypt; manal.hussein@aun.edu.eg (M.T.H.); doaa@aun.edu.eg (D.M.M.); 2 Department of Veterinary Sciences, University of Messina, 98168 Messina, Italy; zacconegiacomo@gmail.com; 3 Department of Chemical, Biological, Pharmaceutical and Environmental Sciences, University of Messina, 98166 Messina, Italy; 4 University School for Advanced Studies—IUSS Pavia, 27100 Pavia, Italy; sebastian.marino@iusspavia.it; 5 Department of Zoology, College of Science, King Saud University, Riyadh 11451, Saudi Arabia; ralonezan@ksu.edu.sa; 6 Department of Anatomy and Histology, School of Veterinary Medicine, Badr University in Assiut, Assiut 19952, Egypt

**Keywords:** fluorescence intensity curve, bio-defense, serotonin signaling, macrophage subsets, telocyte networks, neuroimmune interaction, adaptive immunity

## Abstract

Goldfish (*Carassius auratus*) gills function as both respiratory and immune-regulatory organs, integrating neuroendocrine and immune responses to environmental stimuli. This study explores the spatial organization and interaction of neuroendocrine cells (NECs) and immune cells within goldfish gills using confocal immunohistochemistry and transmission electron microscopy. NECs, identified near blood capillaries and nerve fibers, highlight their role in environmental sensing and physiological regulation. These cells express serotonin (5-HT), a neurotransmitter critical for neuroimmune communication. Two distinct macrophage subsets were observed: iNOS-positive macrophages, concentrated in the basal epithelium, suggest a pro-inflammatory role, whereas 5-HT-positive macrophages, dispersed in the subepithelium, likely contribute to immune modulation. The co-localization of MHC-II and CD68 in macrophages further supports an active antigen-processing system in the gills. Ultrastructural analysis revealed diverse immune cells, including rodlet cells, telocytes, and lymphocytes, within the gill epithelium. Telocytes formed intricate networks with immune cells, highlighting their role in immune coordination and tissue homeostasis. These findings provide new insights into the neuroimmune interactions in fish gills, contributing to a broader understanding of aquatic immune systems and environmental adaptability.

## 1. Introduction

Fish gills have multiple functions, including respiration, osmoregulation, pH regulation, the generation of hormones, and the excretion of nitrogenous waste. Gills are highly specialized organs that house an array of cell types critical for maintaining physiological homeostasis [1]. The diffuse group of cells known as APUD (amine precursor uptake and decarboxylation) cells originate in the neural crest and are the origin of NECs [2]. APUD cells are known to produce substances like enkephalin, dopamine, serotonin, epinephrine, norepinephrine, and cytoplasmic vesicles as well as somatostatin and neurotensin [3]. Neuroendocrine cells (NECs) play a pivotal role in maintaining physiological homeostasis, acting as a crucial link between the nervous and endocrine systems. In teleosts, bioactive compounds generated by NECs are primarily located in the gills, where they are involved in essential processes such as osmoregulation, respiratory regulation, and stress response [4]. The gill filaments of a diverse range of teleost and non-teleost species contain the oxygen sensors of the NECs [5]. Neuroendocrine cells regulate vascular tone in the gills in response to neuronal or stimulatory inputs [6]. Therefore, NECs release neurotransmitters and hormones in response to changes in environmental conditions, particularly those related to water quality, oxygen levels, and ion balance [7,8].

Gills also serve as a crucial immune barrier, protecting fish from external pathogens present in the aquatic environment [9]. As the first point of contact with waterborne pathogens, the gills are equipped with a variety of immune cells that provide both innate and adaptive defense mechanisms [10]. Understanding the role of these immune cells in maintaining fish health is essential, especially for species like goldfish that are often used as model organisms in immunological research.

The goldfish (*Carassius auratus*) is a key biological model in research, valued for its adaptability and well-characterized anatomy. Beyond being popular pets, they serve as essential models for studies in neuroendocrinology, immunology, and histopathology [11]. This study aimed to investigate the detailed histological, immunohistochemical, and ultrastructural characteristics of goldfish gills, with a particular focus on neuroendocrine and immune cell interactions. By examining these cell types and their ultrastructural features, researchers can gain insights into the roles they play in immune surveillance, intercellular communication, and tissue organization.

## 2. Materials and Methods

### 2.1. Sample Collection

The Ethics Committee of Assiut University in Egypt approved the study (ethical number: 06/202/0191). A total of twelve mature healthy goldfish (*Carassius auratus*) were used. Following arrival at the laboratory, the fish were kept for an adaptation period of 2 weeks to monitor their health condition. During this period, individuals that exhibited abnormal appearance and/or behavior were excluded from the study. In addition to behavioral observation, parasitological and bacteriological examinations were performed to support the health screening. For parasitological assessment, wet mount preparations of gill filaments were examined microscopically to detect the presence of external parasites such as *Ichthyophthirius multifiliis*, *Trichodina* spp., and monogenean trematodes [12]. No parasitic infections were observed in the examined samples. Furthermore, bacterial smears were taken from the skin, gills, and intestinal mucosa of randomly selected fish and were stained using the Gram stain technique [13]. Microscopic examination revealed no signs of heavy bacterial growth.

Water quality was carefully maintained during the adaptation and experimental periods: temperature 22 ± 1 °C, dissolved oxygen >6 mg/L, pH 7.5 ± 0.2, and undetectable levels of ammonia and nitrite. Water was partially renewed daily to ensure a stable and clean environment.

The selected fish, averaging 30.50 ± 3.20 g and 9.10 ± 1.0 cm in standard length, were randomly chosen and euthanized with an overdose of MS-222 (3% tricaine) prior to tissue sampling [14]. The gills [6,15] were removed and prepared for histological examination.

### 2.2. Confocal Immunofluorescence

Small tissue samples were taken from goldfish gills and fixed in 4% paraformaldehyde (PFA) in PBS for 3–6 h. Then samples were washed in PBS, dehydrated in increasing concentrations of ethanol, cleared in xylene, embedded in paraffin wax, and serially sectioned. The immunolabeling procedures were comparable to those reported for a broad range of fish organs [6,15]. After being cleaned with distilled water, the sections were placed in a blocking solution with PBS that contained Triton-100 for one hour (Sigma-Aldrich, Saint Louis, MO, USA), 0.02% bovine serum albumin (BSA, Jackson Immunoresearch, West Grove, PA, USA), 1% dimehtyl sulfoxide, 0.02% sodium azide, and 5% normal horse serum (Jackson Immunoresearch, West Grove, PA, USA). Next, the permeabilized tissue sections were incubated with appropriate secondary antibodies at room temperature for 1 h in the dark. Then, a Carl Zeiss LSM 700 confocal laser system (Carl Zeiss AG, Oberkochen, Germany) with solid-state laser lines (405, 488, and 639 nm) was used to investigate the cover-slipped sections, and a variable-spectral secondary beam filter. Using Adobe Photoshop CC Version 2019 (Adobe System, San Jose, CA, USA), the digital images were inserted in a figure composite. The display profile function of Zen 2011 (blue edition 1.0) was then used to assess the fluorescence intensity curves.

Primary antibodies (Table 1); 5-hydroxy- tryptamine (5-HT), inducible nitric oxide synthase (iNOS), peptide-MHC class II, and CD68 were used in double-labeling methods. The specificity, characterization, and reliability of all primary antibodies were previously examined in the skin, gills, and gut of several fish species. The gut and skin were selected for antibody validation due to their rich populations of neuroepithelial cells, dense innervation, and well-developed mucosal immune systems, which make them suitable reference tissues for confirming antibody specificity prior to application in the gills [5,16].

### 2.3. Semithin Sections and TEM Preparations

The gills were fixed by immersion in a 2.5% paraformaldehyde–glutaraldehyde solution for 24 h [17]. After fixation, the samples were rinsed in 0.1 M phosphate buffer and osmicated in 0.1 M sodium-cacodylate buffer (pH 7.3) with 1% osmium tetroxide. Following ethanol and propylene oxide dehydration, the samples were embedded in Araldite. Toluidine blue was used to stain the semithin (1 µm thick) sections that were made with Richert Ultracuts (Leica, Wetzlar, Germany). Using an Ultrotom VRV (LKB Bromma, Giessen, Germany), ultrathin slices (70 nm thick) were cut, stained with uranyl acetate and lead citrate [18], and examined using a JEOL 100CX II transmission electron microscope (JEOL, Tokyo, Japan) at the Electron Microscopy Unit of Assiut University.

## 3. Results

### 3.1. Light Microscopy

**Figure 1:**
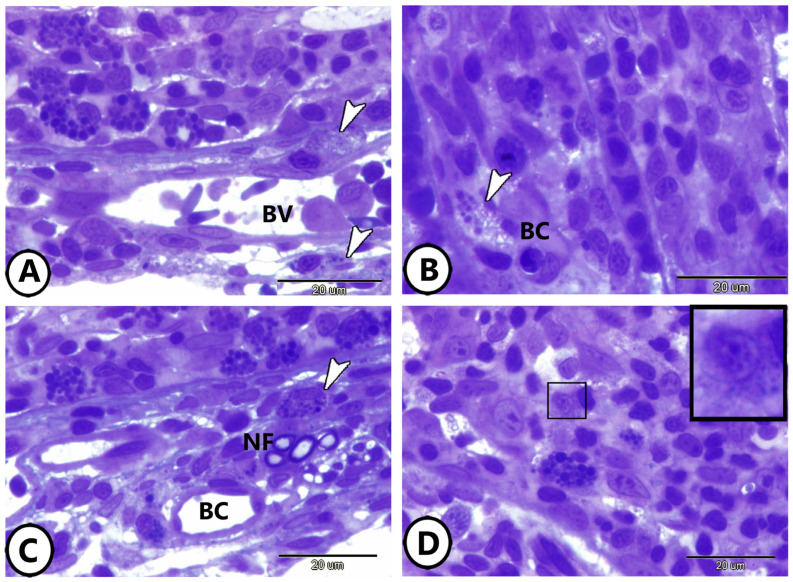
Semithin sections of the cellular components of the gills. (**A**,**B**) Neuroendocrine cells (arrowheads) in direct contact with blood vessels (BV). (**C**) Neuroendocrine cells (arrowhead) in direct contact with nerve fibers (NF) and blood capillaries (BC). (**D**) Neuroendocrine cells (boxed areas) in the epithelium of the primary lamellae of the gills.

Examination of semithin sections of goldfish gill tissues revealed the presence of neuroendocrine cells (NECs) in various structural arrangements. NECs were found in direct contact with blood capillaries (Figure 1A,B), and positioned in direct contact with both nerve fibers and blood capillaries (Figure 1C). Additionally, Figure 1D illustrates the localization of NECs within the epithelium of the primary lamellae.

**Figure 2:**
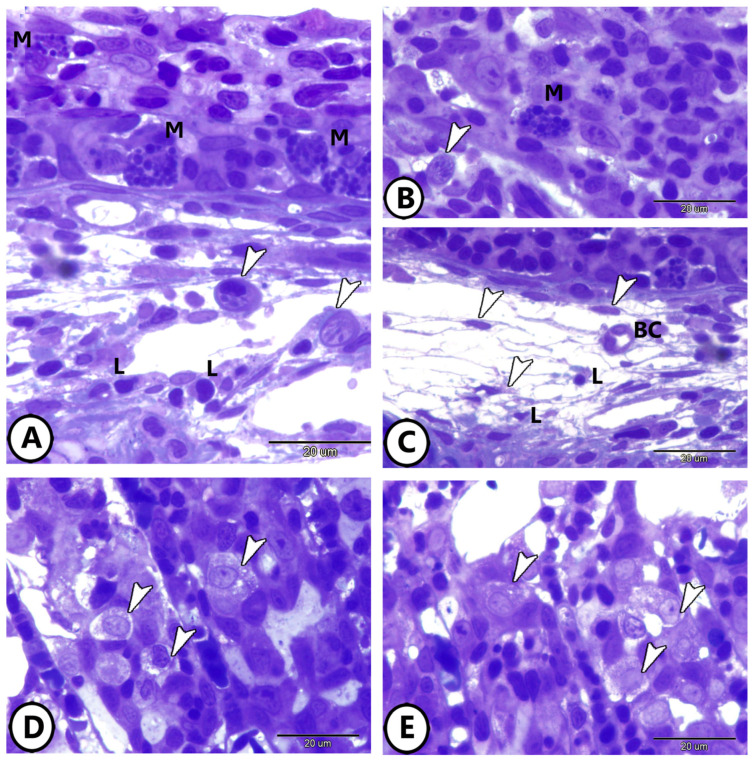
Semithin sections of the immune cells’ components of the gills. (**A**,**B**) Mast cells (M), lymphocytes (L), and different stages of rodlet cells (arrowheads) in the gill tissue. (**C**) Network of Yelocytes (TCs) (arrowheads) in the underlying connective tissue in contact with blood capillaries (BC) and lymphocytes (L). (**D**,**E**) Macrophages (arrowheads) in the epithelium of primary gill lamellae.

Semithin sections also highlighted the presence of diverse immune cells within the gill epithelium. Mast cells and lymphocytes are abundant, and various stages of rodlet cells are observed (Figure 2A,B). Figure 2C depicts a network of telocytes (TCs) within connective tissue in contact with blood capillaries and lymphocytes. Macrophages are prominently featured within the epithelium of the primary gill lamellae (Figure 2D,E).

### 3.2. Confocal Immunohistochemistry

**1.**  **Macrophage Distribution and Labeling (CD68 and MHC II)**

**Figure 3:**
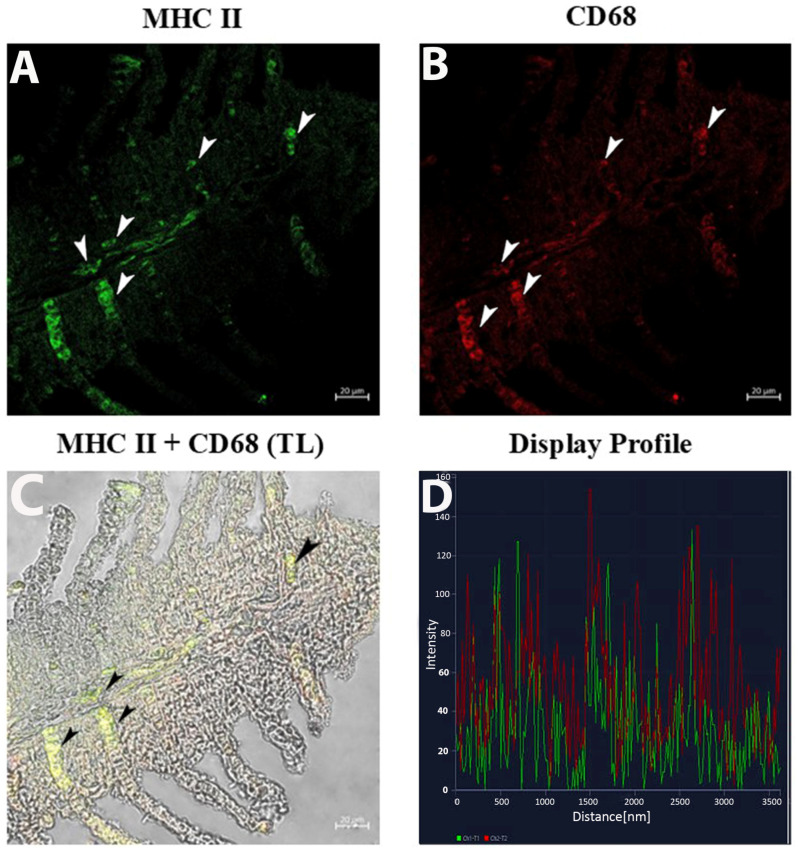
Section of the gill of goldfish, immunofluorescence of MHC II and CD68. (**A**,**B**) MCs positive to MHC II (green) and CD68 (red) can be clearly seen (arrowheads). (**C**) MCs are immunoreactive to CD68 and MHC II (arrowheads). TL: Transmitted light. (**D**) The co-localization of antibodies is confirmed by the ‘display profile’ function. Lines indicate fluorescent signal curves of MHCII (green) and CD68 (red).

The CD68 and MHC II immunolabeling highlight the distinct localization of macrophages in the distal halves of the gill epithelium and subepithelium (Figure 3A,B). CD68 labeling revealed bright fluorescence marking individual macrophages (Figure 3B), while MHC II signals appeared more diffuse, encircling CD68-positive regions (Figure 3A). Double-label overlay images (CD68 + MHC II) emphasized co-localization, particularly in clusters within the basal part of the epithelium. (Figure 3C). The display profile feature verifies the antibodies’ co-localization (Figure 3D).

**2.**  **iNOS Expression**

**Figure 4:**
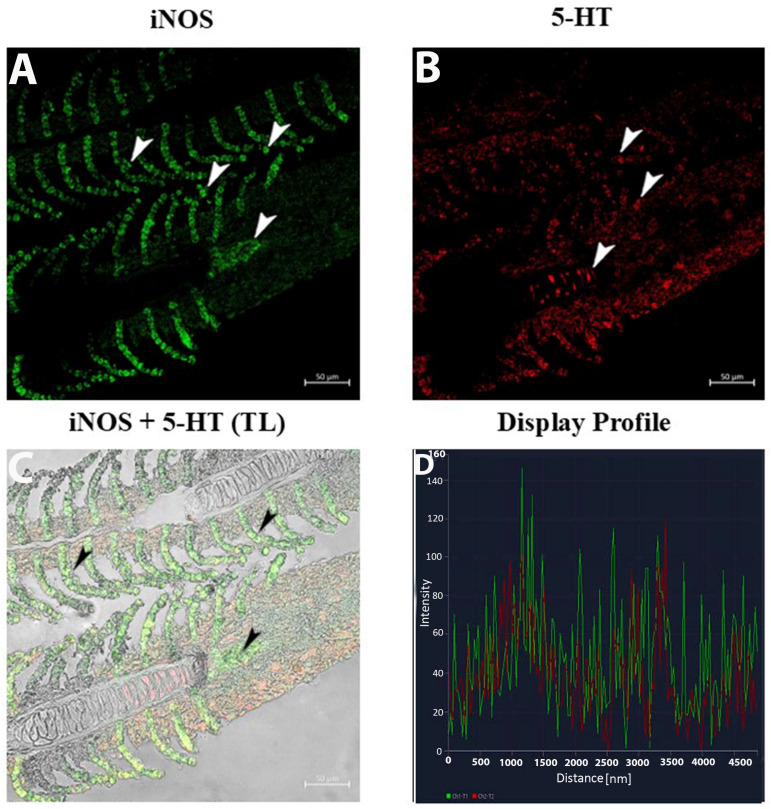
Section of the gill of goldfish, immunofluorescence of iNOS and 5-HT. (**A**,**B**) MCs positive to iNOS (green) and NECs are immunoreactive to 5-HT (red) can be clearly seen (arrowheads). (**C**) MCs are immunoreactive to iNOS and 5-HT (arrowheads). TL: Transmitted light. (**D**) The co-localization of antibodies is confirmed by the ‘display profile’ function. Lines indicate fluorescent signal curves of iNOS (green) and 5-HT (red).

The display profile of iNOS immunoreactivity showed distinct, intense fluorescent signals localized within the macrophage clusters along the epithelium of the gill lamellae. These signals were well-defined and concentrated, suggesting active iNOS expression in specific macrophage subsets. The subepithelial regions exhibited sparse iNOS-positive cells with less clustered distributions (Figure 4A–D).

**3.**  **5-HT Expression in NECs and Macrophages**

**Figure 5:**
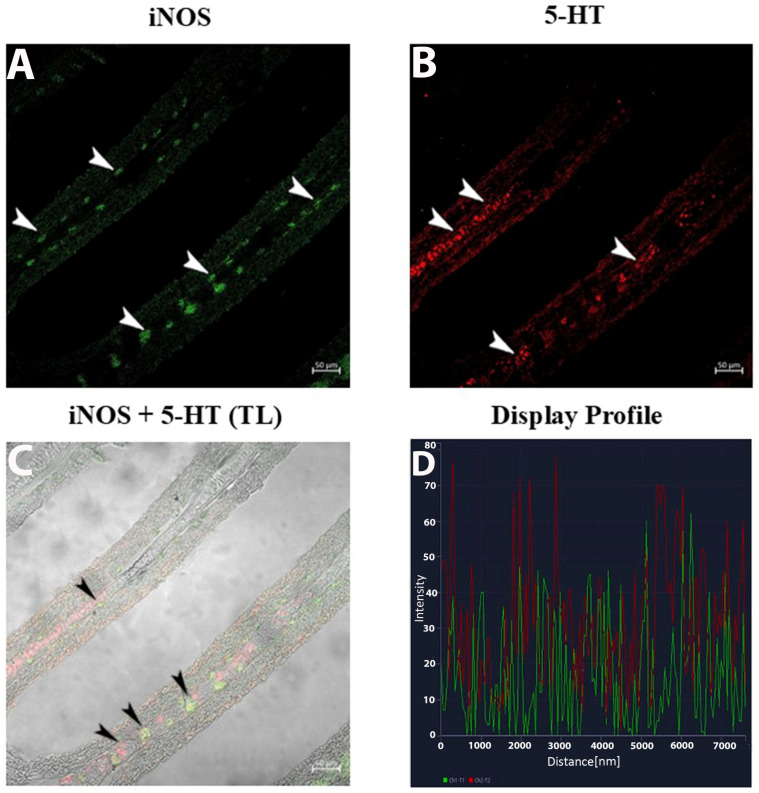
Section of the gill of goldfish, immunofluorescence of iNOS and 5-HT. (**A**,**B**) MCs positive to iNOS (green) and 5-HT (red) can be clearly seen (arrowheads) in the subepithelium. (**C**) Two subsets of MCs are immunoreactive to iNOS and 5-HT (arrowheads). TL: Transmitted light. (**D**) The co-localization of antibodies is confirmed by the ‘display profile’ function. Lines indicate fluorescent signal curves of iNOS (green) and 5-HT (red).

The 5-HT labeling presented as fine, elongated signals correlating with nerve fibers and neuroepithelial cells (NECs) (Figure 4B). Within the subepithelium, 5-HT and iNOS fluorescence identified singular and clustered macrophages (Figure 5B).

**4.**  **Double Immunolabeling for iNOS and 5-HT**

The double-label display profiles for iNOS and 5-HT showcased two non-overlapping macrophage subsets: (Figure 5A–D).

- **iNOS-positive macrophages**: Distinct, bright fluorescence clustered in the basal epithelium, forming compact groups.
- **5-HT-positive macrophages**: Scattered, weaker fluorescence was observed exclusively in the subepithelium.

The use of the display profile function of the confocal microscope supported the current data, highlighting the fluorescence peaks at the individual localization and the co-localization, consistent with the results obtained.

### 3.3. Electron Microscopy

**Figure 6:**
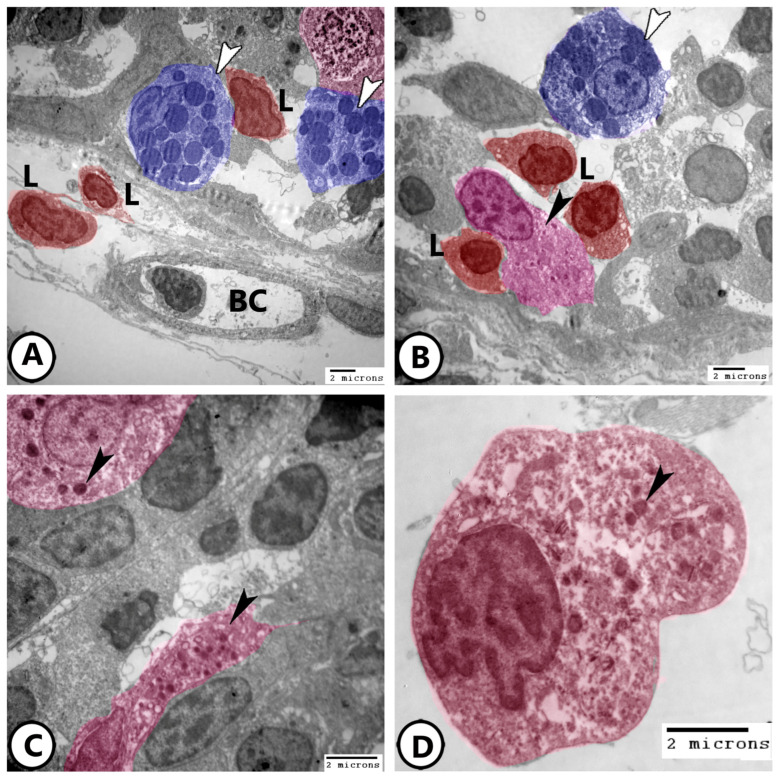
Digitally colored electron EM images of neuroendocrine cells (NECs). (**A**,**B**) Epithelium of the primary lamellae of the gills showing mast cells (blue, white arrowheads), lymphocytes (red, L), NECs (pink, black arrowheads). Note the presence of underlying blood capillaries (BC). (**C**,**D**) Higher magnification of the NECs (pink) showing electron-dense bodies (arrowheads) and many ribosomes.

Ultrastructural analysis provided a detailed visualization of neuroendocrine cells in Figure 6A,B, where mast cells, lymphocytes, and NECs are identified within the epithelium of the primary lamellae. The cytoplasm of NECs contains electron-dense bodies and abundant ribosomes (Figure 6C,D).

**Figure 7:**
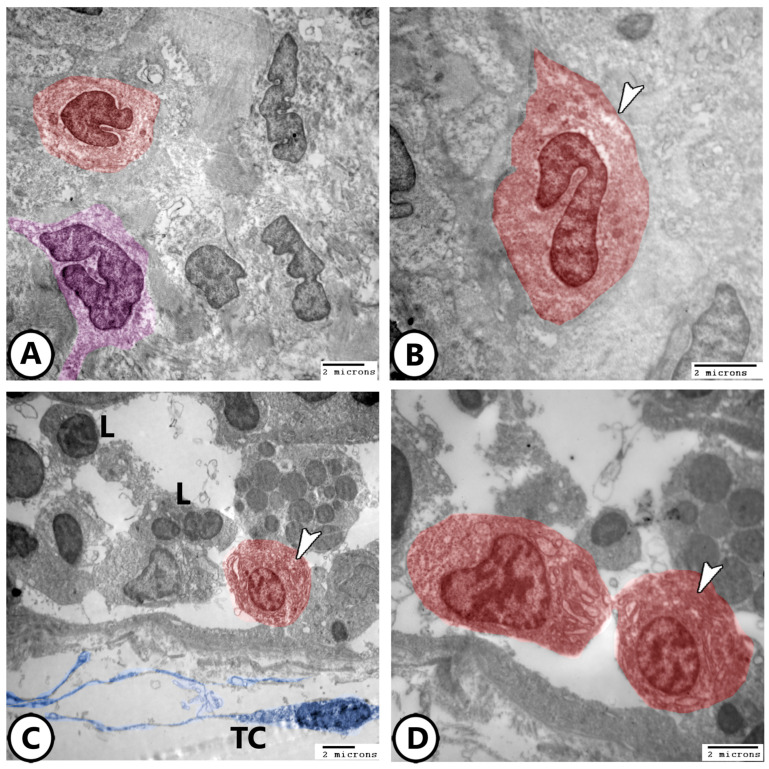
Digitally colored electron EM images of immune cells. (**A**,**B**) Granular leucocytes distributed in the epithelium include eosinophils (red, arrowheads) and neutrophils (violet). (**C**,**D**) Plasma cells (red, arrowheads) are present in the gill epithelium in association with lymphocytes (L). Note the presence of telocytes and their telopodes (blue, TC).

Immune cells are further characterized using electron microscopy as shown in Figure 7A,B, where granular leucocytes, including eosinophils and neutrophils, are distributed throughout the epithelium. Neutrophils are characterized by a segmented nucleus, whereas eosinophils exhibit a bilobed nucleus with rounded cytoplasmic granules. Plasma cells were observed in association with lymphocytes (Figure 7C,D), along with telocytes. The plasma cells exhibited an abundance of rER profiles. Telocytes consist of a cell body containing an oval nucleus and cell processes called telopodes and form a network around immune cells.

**Figure 8:**
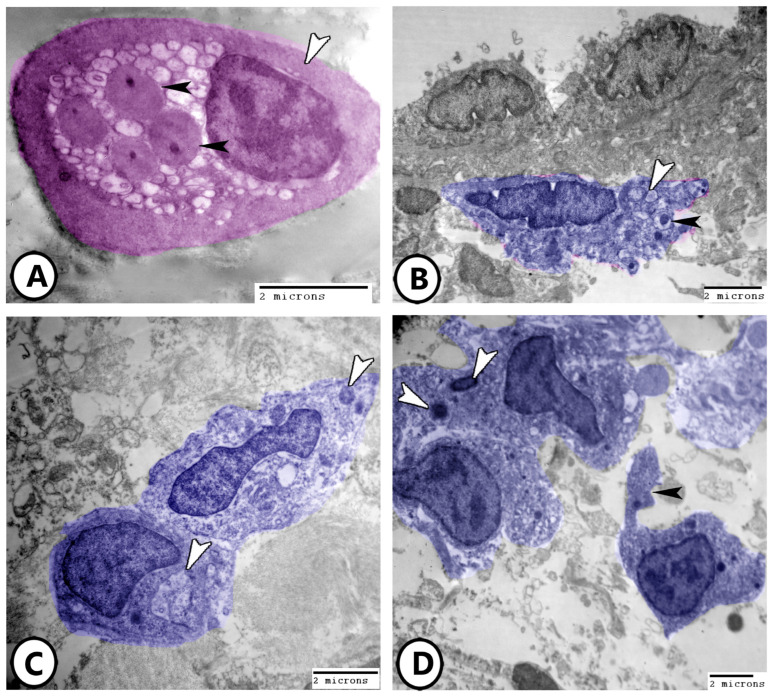
Digitally colored electron EM images of immune cells. (**A**) Rodlet cell (pink) with a characteristic thick capsule (white arrowhead) and rodlet-like inclusions (black arrowheads). (**B**) Macrophage (blue) in the underlying connective tissue containing vacuoles (white arrowhead) and lysosomes (black arrowhead). (**C**,**D**) Macrophages with ingested phagosomes (white arrowheads) and pseudopodia (black arrowhead).

**Figure 9:**
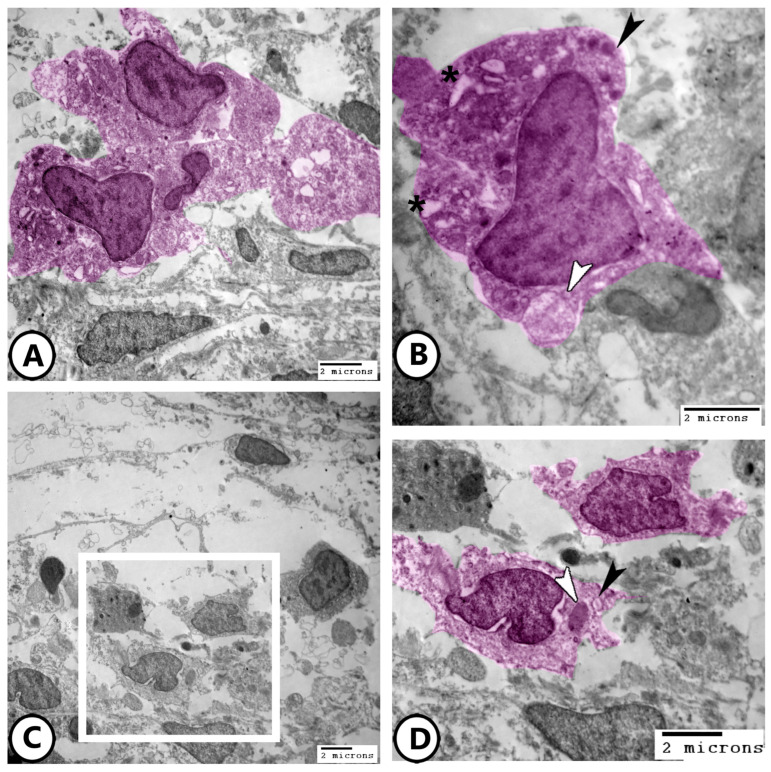
Digitally colored electron EM images of macrophages. (**A**,**B**) Low and higher magnifications of the macrophages (pink) containing phagocytic vacuoles (asterisks), lysosomes (black arrowhead), and membrane-bound vesicles (white arrowhead). (**C**,**D**) Low and higher magnifications of the macrophages (pink, boxed area) containing phagocytosed materials (white arrowhead) and membrane-bound vesicles (black arrowhead).

Rodlet cells are distinctly visualized in Figure 8A, characterized by a thick capsule and rodlet-like inclusions. Macrophages within the connective tissue, containing vacuoles and lysosomes, could be identified (Figure 8B). Figure 8C,D further demonstrate macrophages with ingested phagosomes and pseudopodia. They also possessed phagocytic vacuoles, lysosomes, phagocytosed materials, and membrane-bound vesicles (Figure 9A–D).

**Figure 10:**
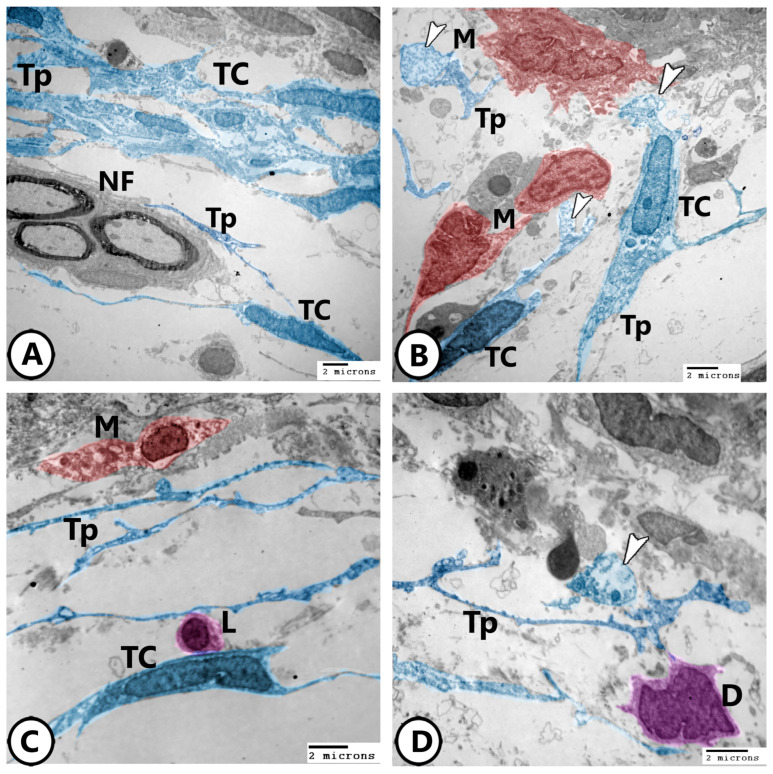
Digitally colored electron EM images of telocytes. (**A**) Network of TCs with their telopodes (TPs) around nerve fibers (NF). (**B**) TCs and their telopodes (TPs), appearing in blue color, in close association to macrophages (red, M). Note the released secretory vesicles (arrowheads) of TCs toward macrophages. (**C**) Heterocellular contact of TCs and their TPs with macrophage (red, M) and lymphocyte (violet, L). (**D**) Heterocellular contact of TCs and their TPs with dendritic cells (violet, D). Note the presence of released secretory vesicles (arrowhead).

Electron microscopy of telocytes as shown in Figure 10A,B display a network of telocytes and their telopodes interacting with nerve fibers and macrophages, respectively. The release of secretory vesicles from telocytes toward macrophages was observed in Figure 10B. The heterocellular contacts of telocytes with macrophages, lymphocytes, and dendritic cells were demonstrated (Figure 10C,D).

## 4. Discussion

The gills and digestive tract of most teleosts, such as carp (*Cyprinus carpio*), goldfish (*Carassius auratus*), pike (*Esox lucius*), and brown trout (*Salmo trutta*), appear to contain mast cells, lymphocytes, and rodlet cells [19,20,21]. These features demonstrate their ability to respond and monitor the immune system actively [22]. A complicated network of immunological interaction and signaling is suggested by the existence of telocytes and their telopodes, as well as plasma cells associated with lymphocytes [23]. A communication role has been suggested by the secretory vesicles that telocytes discharge toward macrophages [24]. The telocytes’ heterocellular interactions with dendritic cells, lymphocytes, and macrophages emphasize their role in tissue homeostasis and cellular communication [25,26]. A network of telocytes in the gills’ connective tissue that interfaces with lymphocytes and blood capillaries suggests that they may play a part in immunological regulation and structural support [27].

The current investigation demonstrated that the gill lamella epithelium contains NECs and their proximity to blood capillaries and nerve fibers indicates a critical function in detecting and reacting to both local and systemic cues. NECs have been shown to be distributed throughout the suprabranchial chamber of the accessory respiratory organs in air-breathing catfish, the gill fans, the lamellae of the gill arches in several teleost species, and the gill filaments of several fish species [28]. The thick core granules observed in NECs are packed with hormones and neuropeptides that either operate locally in the gill tissue or are discharged into the bloodstream. These secretions have an impact on immunological responses, respiratory function, and vascular tone regulation [8].

In *Heteropneustes fossilis*, certain cells of the diffuse neuroendocrine system appear to have oxygen receptors that mediate the cardiovascular and ventilatory response to oxygen shortage [29]. Accordingly, it is thought that solitary neuroendocrine cells play a crucial role in the encouragement and regulation of the airway’s growth and differentiation in a paracrine or autocrine manner [8]. In addition to their endocrine nature and ability to secrete compounds that modulate immunity, these NECs are increasingly recognized as key chemosensory elements in fish, particularly in relation to environmental monitoring and physiological adaptation. Several studies have demonstrated that NECs in the gills are responsive to hypoxic conditions, where they play a role in detecting low oxygen levels and modulating cardiorespiratory reflexes [30,31]. In addition, NECs have been shown to interact with afferent nerve fibers, contributing to neural signaling pathways that regulate ventilation and blood flow [32]. Additionally, NECs of teleost and air-breathing fish displayed immunoreactivity to a range of neuronal markers, including acetylcholine nicotine receptor (AChR), GABA-B-R1 receptor, 5-HT, nitric oxide, catecholamines, and GAD67 [15,33].

In teleost fish, variations in the amount of internal (blood) or external (water) oxygen can cause acute hypoxic responses [34]. These instinctive responses probably originated mainly from the gills’ chemoreceptors being stimulated [35,36,37]. Investigation indicates that NECs, which have dense core vesicles with serotonin (5-HT) as the main monoamine, are the chemoreceptors responsible for these responses. Neurons that innervate serotonergic NECs project to the medulla’s nucleus tractus solitarius (NTS), which is exposed to both efferent branchial blood flow and water flow [7,30]. In fish, NECs are not only involved in maintaining homeostasis under normal conditions and act as critical responders during stress [38].

This study utilized confocal immunohistochemistry to examine the localization and expression profiles of macrophages and neurochemical markers in the gill epithelium and subepithelium of goldfish. The present findings provide insights into the spatial distribution, functional specialization, and heterogeneity of macrophage subsets within this tissue. The expression of 5-HT in nerve fibers and NECs reinforces its role in neuroimmune communication within the gill [39]. To further strengthen their function in preserving tissue integrity and organismal health, NECs work closely with immune cells, including lymphocytes and macrophages to coordinate immune responses [40].

Macrophages are found in the epithelium of the goldfish’s major gill lamellae, emphasizing their function in immunological defense and phagocytosis. MHC class II genes have a direct functional association to immune responses and are constitutively expressed in antigen-presenting cells such dendritic cells, B cells, monocytes, and macrophages [41,42]. Since the gills are an organ with a high T cell abundance, the presence of MHC class II expression points to an additional function for antigen presentation [43,44]. The MHC class II B cDNAs have been detected in several teleost species, such as carp [45], zebrafish (*Danio rerio*) [46], channel catfish (*Ictalurus punctatus*), and salmonid [47,48]. The MHC II genes are highly expressed in the head kidney, intestine, gill, stomach, heart, and spleen, while their expression is low in muscle and blood in nonchallenged red sea bream. These genes are thus associated with tissues that typically contain high concentrations of lymphoid or myeloid cells [49]. Similarly, CD68, a highly glycosylated type I transmembrane glycoprotein, shares structural similarities with the lysosome-associated membrane protein (LAMP) family of glycoproteins [50]. Moreover, CD68 is predominantly located in the endosomal/lysosomal compartments of macrophages [51]. To date, CD68 has been studied mainly in mammals and rarely in fish [52]. The expression of CD68 was much higher in the head kidney, spleen, liver, and heart than in other healthy tissues under normal physiological conditions [53,54]. The co-localization of MHC-II and CD68 in the gills indicates an active immune response in the gills by secreting cytokines and playing a part in the phagocytosis of invasive pathogens.

Interestingly, the present investigation revealed that 5-HT-positive macrophages were restricted to the subepithelium and displayed dispersed distribution. This pattern suggests a modulatory role of 5-HT in immune–neuronal crosstalk, rather than direct involvement in localized immune activation [55]. The absence of 5-HT-positive macrophages in the basal epithelium further supports the notion of specialized functional roles among macrophage subsets.

In the cuurent results, the strong, concentrated iNOS signals observed in macrophage clusters suggest active nitric oxide synthesis, a marker of macrophage activation [56,57]. The functional implications of this localized activation are likely related to both immune defense and modulation of local physiological processes [58]. In contrast, sparse iNOS-positive cells in subepithelial regions may represent a less activated or alternative macrophage phenotype, emphasizing functional heterogeneity within this immune cell population. The gills of *H. fossilis* have previously shown that environmental stimuli trigger the release of NO through a paracrine mechanism for local modulation of muscle tone [59]. Remarkably, it has been observed that the NECs found in fish gills have similarities in their morphology with those of other peripheral O2 chemoreceptors, such as the lungs of lungfish and bichirs, as well as the mammalian carotid body [6].

The distinct fluorescence patterns of iNOS- and 5-HT-positive macrophages imply the coexistence of two non-overlapping subsets. iNOS-positive macrophages clustered in the basal epithelium, indicating a robust pro-inflammatory role [60,61]. In contrast, 5-HT-positive macrophages were scattered in the subepithelium, consistent with their more regulatory or modulatory function [62,63]. This segregation aligns with the concept of macrophage plasticity, in which local microenvironmental cues shape functional differentiation.

## 5. Conclusions

This study highlights the intricate interplay between neuroendocrine and immune cells in the gills of goldfish. The spatial organization and functional diversity of these cells emphasize their critical roles in environmental sensing, immune defense, and physiological regulation. The distinct macrophage subsets and their interactions with telocytes and neuroendocrine cells suggest specialized mechanisms for maintaining gill homeostasis. These findings enhance the understanding of fish immunity and stress responses, offering valuable insights for aquatic biology and environmental adaptation studies.

## Figures and Tables

**Table 1:** Antibodies used and working dilutions.

Antibody	Supplier	Dilution	Animal Source
5-HT	Sigma-Aldrich, Saint Louis, MO, USA (S5545)	1:2000	Rabbit
Inos	Santa Cruz Biotechnology, Inc., Dallas, TX, USA	1:200	Mouse
CD68	Thermo Fisher Scientific, Waltham, MA, USA	1:200	Rabbit
MHC II	Santa Cruz Biotechnology, Inc., Dallas, TX, USA	1:200	Mouse
Alexa Fluor 488 Donkey anti-MouseIgG FITC conjugated	Molecular Probes, Invitrogen, Eugene, OR, USA	1:300	Donkey
Alexa Fluor 594 Donkey anti-RabbitIgG TRITC conjugated	Molecular Probes, Invitrogen, Eugene, OR, USA	1:300	Donkey

## Data Availability

The data presented in this study are available within the article.

## References

[1] Evans D.H., Piermarini P.M., Choe K.P. (2005). The multifunctional fish gill: Dominant site of gas exchange, osmoregulation, acid-base regulation, and excretion of nitrogenous waste. Physiol. Rev..

[2] Pearse A. (1969). The cytochemistry and ultrastructure of polypeptide hormone-producing cells of the APUD series and the embryologic, physiologic and pathologic implications of the concept. J. Histochem. Cytochem..

[3] Cutz E. (1982). Neuroendocrine cells of the lung an overview of morphologic characteristics and development. Exp. Lung Res..

[4] Kotsyuba E., Dyachuk V. (2023). Role of the neuroendocrine system of marine bivalves in their response to hypoxia. Int. J. Mol. Sci..

[5] Porteus C.S., Pollack J., Tzaneva V., Kwong R.W., Kumai Y., Abdallah S.J., Zaccone G., Lauriano E.R., Milsom W.K., Perry S.F. (2015). A role for nitric oxide in the control of breathing in zebrafish (*Danio rerio*). J. Exp. Biol..

[6] Zachar P.C., Jonz M.G. (2012). Neuroepithelial cells of the gill and their role in oxygen sensing. Respir. Physiol. Neurobiol..

[7] Bailly Y., Dunel-Erb S., Laurent P. (1992). The neuroepithelial cells of the fish gill filament: Indolamine-immunocytochemistry and innervation. Anat. Rec..

[8] Zaccone G., Mauceri A., Fasulo S., Ainis L., Lo Cascio P., Ricca M.B. (1996). Localization of immunoreactive endothelin in the neuroendocrine cells of fish gill. Neuropeptides.

[9] Goss G.G., Perry S.F., Fryer J.N., Laurent P. (1998). Gill morphology and acid-base regulation in freshwater fishes. Comp. Biochem. Physiol. A Mol. Integr. Physiol..

[10] Zhou T., Fang Z., Duarte D.F.C., Fernandes S.A., Lu Y., Guo J., Gui L., Chen L. (2022). Transcriptome Analysis of Immune Response against *Streptococcus agalactiae* Infection in the Nile Tilapia GIFT Strain. Fishes.

[11] Simmons D., McCallum E., Balshine S., Chandramouli B., Cosgrove J., Sherry J.P. (2017). Reduced anxiety is associated with the accumulation of six serotonin reuptake inhibitors in wastewater treatment effluent exposed goldfish *Carassius auratus*. Sci. Rep..

[12] Noga E.J. (2010). Fish Disease: Diagnosis and Treatment.

[13] Austin B., Austin D.A. (2016). Bacterial Fish Pathogens: Disease of Farmed and Wild Fish.

[14] Posner L.P., Scott G.N., Law J.M. (2013). Repeated exposure of goldfish (*Carassius auratus*) to tricaine methanesulfonate (MS-222). J. Zoo Wildl. Med..

[15] Zaccone G., Mauceri A., Fasulo S. (2006). Neuropeptides and nitric oxide synthase in the gill and the air-breathing organs of fishes. J. Exp. Zool. A Comp. Exp. Biol..

[16] Maina J.N., Icardo J.M., Zaccone G., Aragona M., Lauriano E.R., Alesci A., Albano M., Guerrera M.C., Germana A., Fernandes J.M.O. (2022). Immunohistochemical and ultrastructural study of the immune cell system and epithelial surfaces of the respiratory organs in the bimodally breathing African sharptooth catfish *(Clarias gariepinus* Burchell, 1822). Anat. Rec..

[17] Karnovsky M. (1965). A Formaldehyde-Glutaraldehyde Fixative of High Osmolality for Use in Electron Microscopy. J. Cell Biol..

[18] Reynolds E.S. (1963). The use of lead citrate at high pH as an electron-opaque stain in electron microscopy. Cell Biol..

[19] Chiu H., Lagunoff D. (1972). Histochemical comparison of vertebrate mast cells. Histochem. J..

[20] Temkin R.J., McMillan D.B. (1986). Gut-associated lymphoid tissue (GALT) of the goldfish, *Carassius auratus*. J. Morphol..

[21] Chiarini-Garcia H., Ferreira R. (1992). Histochemical evidence of heparin in granular cells of *Hoplias malabaricus* Bloch. Fish Biol..

[22] Reite O.B., Evensen Ø. (2006). Inflammatory cells of teleostean fish: A review focusing on mast cells/eosinophilic granule cells and rodlet cells. Fish Shellfish Immunol..

[23] Vannucchi M.G. (2020). The Telocytes: Ten Years after Their Introduction in the Scientific Literature. An Update on Their Morphology, Distribution, and Potential Roles in the Gut. Int. J. Mol. Sci..

[24] Zhao J., Birjandi A.A., Ahmed M., Redhead Y., Olea J.V., Sharpe P. (2022). Telocytes regulate macrophages in periodontal disease. eLife.

[25] Verdile N., Pasquariello R., Cardinaletti G., Tibaldi E., Brevini T.A.L., Gandolfi F. (2022). Telocytes: Active Players in the Rainbow Trout (*Oncorhynchus mykiss*) Intestinal Stem-Cell Niche. Animals.

[26] Aleksandrovych V., Gil A., Poniatowski A. (2022). Notes about telocytes and immunity. Folia Med. Cracov..

[27] Mokhtar D.M., Hussein M.M., Sayed R.K. (2022). Novel identification and microscopy of the intestinal bulb of molly fish (*Poecilia sphenops*) with a focus on its role in immunity. Microsc. Microanal..

[28] Zaccone G., Lauriano E.R., Capillo G., Kuciel M. (2018). Air-breathing in fish: Air-breathing organs and control of respiration: Nerves and neurotransmitters in the air-breathing organs and the skin. Acta Histochem..

[29] Mauceri A., Fasulo S., Ainis L., Licata A., Lauriano E.R., Martínez A., Mayer B., Zaccone G. (1999). Neuronal nitric oxide synthase (nNOS) expression in the epithelial neuroendocrine cell system and nerve fibers in the gill of the catfish, *Heteropneustes fossilis*. Acta Histochem..

[30] Jonz M.G., Fearon I.M., Nurse C.A. (2004). Neuroepithelial oxygen chemoreceptors of the zebrafish gill. J. Physiol..

[31] Qin Z., Lewis J.E., Perry S.F. (2010). Zebrafish (*Danio rerio*) gill neuroepithelial cells are sensitive chemoreceptors for environmental CO_2_. J. Physiol..

[32] Jonz M.G., Nurse C.A. (2003). Neuroepithelial cells and associated innervation of the zebrafish gill: A confocal immunofluorescence study. J. Comp. Neurol..

[33] Porteus C.S., Brink D.L., Coolidge E.H., Fong A.Y., Milsom W.K. (2013). Distribution of acetylcholine and catecholamines in fish gills and their potential roles in the hypoxic ventilatory response. Acta Histochem..

[34] Randall D. (1982). The control of respiration and circulation in fish during exercise and hypoxia. Exp. Biol..

[35] Dunel-Erb S., Bailly Y., Laurent P. (1982). Neuroepithelial cells in fish gill primary lamellae. J. Appl. Physiol..

[36] Sundin L., Nilsson G.E., Block M., Löfman C.O. (1995). Control of gill filament blood flow by serotonin in the rainbow trout, *Oncorhynchus mykiss*. Am. J. Physiol. Regul. Integr. Comp. Physiol..

[37] Wilson J.M., Laurent P. (2002). Fish gill morphology: Inside out. Exp. Zool..

[38] Nardocci G., Navarro C., Cortés P.P., Imarai M., Montoya M., Valenzuela B., Jara P., Acuña-Castillo C., Fernández R. (2014). Neuroendocrine mechanisms for immune system regulation during stress in fish. Fish Shellfish Immunol..

[39] Alesci A., Capillo G., Mokhtar D.M., Fumia A., D’Angelo R., Lo Cascio P., Albano M., Guerrera M.C., Sayed R.K.A., Spanò N. (2022). Expression of Antimicrobic Peptide Piscidin1 in Gills Mast Cells of Giant Mudskipper *Periophthalmodon schlosseri* (Pallas, 1770). Int. J. Mol. Sci..

[40] Lauriano E.R., Capillo G., Icardo J.M., Fernandes J.M.O., Kiron V., Kuciel M., Zuwala K., Guerrera M.C., Aragona M., Germana’ A. (2021). Neuroepithelial cells (NECs) and mucous cells express a variety of neurotransmitters and neurotransmitter receptors in the gill and respiratory air-sac of the catfish *Heteropneustes fossilis* (Siluriformes, Heteropneustidae): A possible role in local immune defence. J. Zool..

[41] Beck S., Kelly A., Radley E., Khurshid F., Alderton R.P., Trowsdale J. (1992). DNA sequence analysis of 66 kb of the human MHC class II region encoding a cluster of genes for antigen processing. J. Mol. Biol..

[42] Glimcher L.H., Kara C.J. (1992). Sequences and factors: A guide to MHC class-II transcription. Annu. Rev. Immunol..

[43] Haugarvoll E., Bjerkås I., Nowak B.F., Hordvik I., Koppang E.O. (2008). Identification and characterization of a novel intraepithelial lymphoid tissue in the gills of Atlantic salmon. J. Anat..

[44] Koppang E.O., Fischer U., Moore L., Tranulis M.A., Dijkstra J.M., Köllner B., Aune L., Jirillo E., Hordvik I. (2010). Salmonid T cells assemble in the thymus, spleen and in novel interbranchial lymphoid tissue. J. Anat..

[45] van Erp S.H., Egberts E., Stet R.J. (1996). Characterization of class II A and B genes in a gynogenetic carp clone. Immunogenetics.

[46] Ono H., O’hUigin C., Vincek V., Klein J. (1993). Exon-intron organization of fish major histocompatibility complex class II B genes. Immunogenetics.

[47] Godwin U.B., Flores M., Quiniou S., Wilson M.R., Miller N.W., Clem L.W., McConnell T.J. (1997). MHC class II B genes in the channel catfish (*Ictalurus punctatus*). Dev. Comp. Immunol..

[48] Hardee J.J., Godwin U., Benedetto R., McConnell T.J. (1995). Major histocompatibility complex class II A gene polymorphism in the striped bass. Immunogenetics.

[49] Chen S.-L., Zhang Y.X., Xu M.Y., Ji X.S., Yu G.C., Dong C.F. (2006). Molecular polymorphism and expression analysis of MHC class II B gene from red sea bream (*Chrysophrys major*). Develop. Comp. Immunol..

[50] Holness C.L., da Silva R.P., Fawcett J., Gordon S., Simmons D.L. (1993). Macrosialin, a mouse macrophage-restricted glycoprotein, is a member of the lamp/lgp family. Biol. Chem..

[51] Fukuda M. (1991). Lysosomal membrane glycoproteins. Structure, biosynthesis, and intracellular trafficking. Biol. Chem..

[52] Cui H., Li H., Zhang M., Li H., Wang X., Wang Z., Zhai W., Chen X., Cheng H., Xu J. (2022). Molecular characterization, expression, evolutionary selection, and biological activity analysis of CD68 gene from *Megalobrama amblycephala*. Int. J. Mol. Sci..

[53] Kumar R., Joy K., Singh S. (2016). Morpho-histology of head kidney of female catfish Heteropneustes fossilis: Seasonal variations in melano-macrophage centers, melanin contents and effects of lipopolysaccharide and dexamethasone on melanins. Fish Physiol. Biochem..

[54] Press C.M., Dannevig B., Landsverk T. (1994). Immune and enzyme histochemical phenotypes of lymphoid and nonlymphoid cells within the spleen and head kidney of Atlantic salmon (*Salmo salar* L.). Fish Shellfish Immunol..

[55] Khan N.A., Deschaux P. (1997). Role of serotonin in fish immunomodulation. Exp. Biol..

[56] McNeill E., Crabtree M.J., Sahgal N., Patel J., Chuaiphichai S., Iqbal A.J., Hale A.B., Greaves D.R. (2015). Channon KM. Regulation of iNOS function and cellular redox state by macrophage Gch1 reveals specific requirements for tetrahydrobiopterin in NRF2 activation. Free Radic. Biol. Med..

[57] Nathan C.F., Hibbs J.B. (1991). Role of nitric oxide synthesis in macrophage antimicrobial activity. Curr. Opin. Immunol..

[58] Wink D., Hines H.B., Cheng R.Y., Switzer C.H., Flores-Santana W., Vitek M.P., Ridnour L.A., Colton C.A. (2011). Nitric oxide and redox mechanisms in the immune response. J. Leukoc. Biol..

[59] Martínez A. (1995). Nitric oxide synthase in invertebrates. Histochem. J..

[60] Xue Q., Yan Y., Zhang R., Xiong H. (2018). Regulation of iNOS on immune cells and its role in diseases. Int. J. Mol. Sci..

[61] Hou S.-M., Yang C.-M., Huang W.-C., Cheng S.-W., Yen T.-L., Hsia C.-W., Hsieh C.-Y., Hsia C.-H. (2025). Glabridin Suppresses Macrophage Activation by Lipoteichoic Acid In Vitro: The Crucial Role of MAPKs-IL-1β-iNOS Axis Signals in Peritoneal and Alveolar Macrophages. Biomolecules.

[62] Li Q., Jiang B., Zhang Z., Huang Y., Xu Z., Chen X., Hou X., Cai J., Huang Y., Jian J. (2022). Serotonin system is partially involved in immunomodulation of Nile tilapia (*Oreochromis niloticus*) immune cells. Front. Immunol..

[63] Quintero-Villegas A., Valdés-Ferrer S.I. (2020). Role of 5-HT_7_ receptors in the immune system in health and disease. Mol. Med..

